# 

**Published:** 1908-06-27

**Authors:** 


### NEW REGULATIONS AS TO UNSOUND FOOD AND FOREIGN MEAT.

The Public Health (Regulations as to Food) Act, passed last session, empowered the Local Government Board to make regulations with respect to the importation, preparation and distribution of food. Draft regulations dealing with unsound food and with foreign meat have now been made and issued by the Board. Sanitary authorities and their medical officers of health will receive under these regulations wider and more stringent powers of inspection, especially in the case of articles of food imported into this country. The regulations also strike at an evil which was demonstrated in recent reports by Dr. G. S. Buchanan and Dr. A. W. MacFadden, of the Local Government Board, to be very serious—namely, the importation of large quantities of boned meats, kidneys, tongues, and the like, heavily charged with boric and other preservatives, and also of unwholesome tripe. Officers of Customs are to ascertain whether cargoes of ships entering their district include any foreign meat of certain defined classes, and, if so, the medical officer of health is to examine such meat and may, if he thinks fit, forbid its removal from the place of landing for any purpose other than exportation. Certain rules are laid down for the guidance of medical officers, and provision is made for "destruction orders" by justices of the peace. On the whole, the regulations seem to be so drawn as to secure quite effectively their excellent purpose.

### THE GRADUATES' MEDICAL SCHOOLS.

DIARY FOR WEEK JUNE 29 TO JULY 4.

### ROYAL COLLEGE OF PHYSICIANS, Pall Mall East.

Croonian Lectures.

**At 5 p.m.**

**June 30,** Dr. A. E. Garrod, **Inborn Errors in Metabolism.**

### LONDON HOSPITAL MEDICAL COLLEGE, Turner Street, Mile End, E.

**At 3 p.m.**
**June 30,** Mr. Jonathan Hutchinson, F.R.S., **Vegetable Pathology.**

### NATIONAL HOSPITAL FOR THE PARALYSED AND EPILEPTIC, Queen Sq., Bloomsbury, W.C.

**At 3.30 p.m.**
**June 30,** Mr. Ballance, **Surgery of the Nervous System.**
**July 3,** Dr. A. Turner, **Treatment of Epilepsy.**

### MEDICAL GRADUATES' COLLEGE AND POLYCLINIC, Chenies Street, W.C.

**At 5.15 p.m.**
**June 29,** Dr. G. A. Sutherland, **Acute Abdominal Attacks in Children.**
**June 30,** Dr. T. Claye Shaw, **The Psychology of Failure and Success.**
**July 1,** Mr. E. M. Corner, **Modern Treatment of Fractures—The Arm.**
**July 2,** Mr. E. M. Corner, **Modern Treatment of Fractures—The Leg.**

### THE POST GRADUATE COLLEGE, West London Hospital, Hammersmith, W.

**At 12 noon.**
**June 29,** Dr. Low, **Pathological Demonstration.**
**At 12.15 p.m.**
**July 1 and 3,** Dr. Pritchard, **Practical Medicine.**
**At 5 p.m.**
**June 29,** Mr. R. W. Lloyd, **Anæsthetics—I.**
**June 30,** Mr. Pardoe, **Some Practical Points in the Surgery of the Prostate.**
**July 1,** Dr. Beddard, **Medicine—V.**
**July 2,** Mr. Keetley, **Clinical Lecture—I.**
**July 3,** Dr. Seymour Taylor, **Malingering.**

### THE HOSPITAL FOR SICK CHILDREN, Great Ormond Street, W.C.

**At 4 p.m.**
**July 2,** Dr. Thompson, **Hydrocephalus.**

### LONDON SCHOOL OF CLINICAL MEDICINE, Seamen's Hospital, Greenwich, S.E.

**At 2.30 p.m.**

**July 2,** Dr. Rankin, **Myxœdema.**

### THE THROAT HOSPITAL, Golden Square, W.

**At 5.30 p.m.**
**June 29,** Mr. Faulder, **Chronic Inflammatory Affections of the Larynx.**

### NORTH-EAST LONDON POST-GRADUATE COLLEGE, Prince of Wales's General Hospital, Tottenham, N.

**At 4.30 p.m.**

**June 29,** Dr. T. R. Whipham, **Selected Cases of Disease in Children.**

### CHARING CROSS HOSPITAL, W.C.

Post Graduate Lectures.

**At 4 p.m.**
**July 2,** Dr. MacLeod, **Dermatology.**

### MOUNT VERNON HOSPITAL, Hampstead, N.W.

**At 5 p.m.**
**July 2,** Dr. T. D. Lister, **Cases of Chest Disease in the Wards.**

### THE INCORPORATED SOCIETY OF MEDICAL OFFICERS OF HEALTH.

An extraordinary general meeting of the Incorporated Society of Medical Officers of Health took place at the offices of the Society, 1 Upper Montague Street, Russell Square, London, W.C., on June 19. The meeting was presided over by Dr. George Reid, M.O.H. to the Staffordshire County Council and President of the Society. Resolutions were passed unanimously by which the membership of the Society will be limited in future to two classes—viz., Fellows and Associates. It was also agreed that the Fellowship of the Society shall be open to the following :—Medical officers of health, acting or retired, whether in the British Isles and Dependencies or elsewhere; medical officers (sanitary) of the Navy and Army (at home or abroad); all registered medical practitioners holding a diploma in sanitary science, public health, or State medicine under Section 21 of the Medical Act, 1886; all medical inspectors of the Local Government Board or other Government Departments of England and Wales, Scotland, and Ireland; and medical officers appointed by education authorities. The confirmatory meeting will be held in the Law Courts at Cardiff on July 17, 1908.

THE HOSPITAL *June* 27, 1908.

*Name* ..................................................................

*Address* ..................................................................

..................................................................

This Coupon must accompany manuscript or contributions intended for The Hospital.

